# Higher disease reactivation risk in women after fingolimod withdrawal

**DOI:** 10.1186/s40478-026-02343-6

**Published:** 2026-06-17

**Authors:** Marine Massy, Stefanie Marti, Adriane Kutllovci, Niklas Frahm, Firas Fneish, David Ellenberger, Helly Hammer, Andrew Chan, Alexander Leichtle, Fred Lühder, Imke Metz, Maximilian Pistor, Robert Hoepner

**Affiliations:** 1 Department of Neurology, Inselspital, Bern University Hospital and University of Bern, Freiburgstrasse 16, 3010 Bern, Switzerland; 2 Graduate School for Cellular and Biomedical Sciences, University of Bern, Bern, Switzerland; 3 Institute of Neuropathology, University Medical Center Göttingen, Göttingen, Germany; 4 German MS Register, MS Forschungs- Und Projektentwicklungs-gGmbH (MS Research and Project Development gGmbH (MSFP)), Hannover, Germany; 5 Laboratory Medicine, Cantonal Hospital Baden, Baden, Switzerland; 6 Center for Artificial Intelligence in Medicine, University of Bern, Bern, Switzerland; 7 Institute for Neuroimmunology and Multiple Sclerosis Research, University Medical Center Göttingen, Göttingen, Germany

**Keywords:** Multiple sclerosis, Disease reactivation, S1PRM, Sex-specific, Fingolimod, Sex differences

## Abstract

**Background:**

Disease reactivation following cessation of sphingosine 1-phosphate receptor modulators (S1PRM) occurs in ~ 10% of multiple sclerosis (MS) patients. The biological factors underlying this phenomenon remain incompletely understood, including the potential contribution of sex-specific differences.

**Methods:**

We performed a systematic review on published literature and adverse event registries (FAERS, EudraVigilance), as of January 2024, focusing exclusively on fingolimod (FTY) withdrawal in individuals with MS. Disease severity after FTY withdrawal was assessed in the experimental autoimmune encephalomyelitis (EAE) mouse model, using untreated EAE mice as controls. S1P receptor expression was analyzed by immunofluorescence in spinal cord tissue from EAE mice and in brain biopsies from MS patients with disease reactivation after FTY withdrawal and MS controls (no prior FTY or S1PRM treatment).

**Results:**

Analysis of eight studies (n = 2579) demonstrated an association between female sex and disease reactivation after FTY cessation (odds ratios: 1.09–7.20), corroborated by pharmacovigilance data (FAERS: OR = 2.00, *p* < 0.0001; EudraVigilance: OR = 2.42, *p* < 0.0001). Female EAE mice exhibited greater post-FTY treatment disease severity (2.5-fold increase, *p* < 0.0001) with increased S1PR1 expression on CD3^+^T cells (*p* < 0.001). Human brain biopsies showed elevated S1PR1 expression on CD3^+^T cells in active demyelinating lesions during disease reactivation compared to inactive demyelinated lesions (*p* < 0.0001) and controls (*p* < 0.05).

**Discussion:**

Across clinical, experimental, and neuropathological analyses, female sex was associated with more pronounced disease activity following FTY withdrawal. Increased S1PR1 expression in T cells represents a potential cellular correlate of this sex-associated vulnerability and warrants further mechanistic investigation.

**Supplementary Information:**

The online version contains supplementary material available at 10.1186/s40478-026-02343-6.

## Introduction

Multiple sclerosis (MS) is a chronic, immune-mediated inflammatory disease affecting the central nervous system (CNS), characterized by demyelination and neurodegeneration [20]. With a global prevalence of approximately 2.9 million people as of 2023, MS is a significant worldwide health challenge [50]. The disease manifests through a wide array of symptoms, including fatigue, motor weakness, sensory disturbances, and cognitive impairments, potentially leading to severe disability over time [17]. MS predominantly affects young adults, with a higher prevalence among women (women-to-men ratio up to 4:1 in some countries) [63].

The pathophysiology of MS is complex, involving an interplay between genetic susceptibility and environmental factors that trigger an abnormal immune response [32]. This response leads to inflammation, demyelination, and neurodegeneration in the CNS. T cells, along with B cells, play a crucial role in this process, with lymphocytes being among the most frequent cell types found in MS lesion infiltrates, second only to macrophages [26].

Sphingosine 1-phosphate receptor modulators (S1PRMs) have emerged as a promising class of treatments for MS due to their impact on the immune system, particularly on lymphocyte migration [7]. By binding to S1P receptors on lymphocytes and inhibiting their egress from lymphoid tissues into the circulation, S1PRMs effectively reduce CNS inflammation [37]. These disease modifying therapies have demonstrated significant effects in reducing relapses and disability progression in people with MS (pwMS) [14].

Fingolimod (FTY), an S1PRM, was the first oral treatment approved for treating relapsing–remitting MS (RRMS). It has shown substantial efficacy in reducing disease activity and slowing disability progression in several clinical trials [40]. FTY acts by non-selectively targeting S1PR1, 3, 4 and 5 receptors on lymphocyte surfaces, modulating immune responses and preventing autoreactive T cells from infiltrating the CNS [8]. Differential S1P receptor expression on specific lymphocyte subsets, such as CD4+T cells and CD19+B cells, may help explain individual susceptibility to FTY discontinuation-related disease reactivation [6]. Studies have described divergent dynamics of lymphocyte subset reconstitution following treatment cessation [30], potentially driven by variable regulation of S1PR1-mediated signaling, which may underlie the risk of disease exacerbation after treatment withdrawal. This variability could influence the individual risk of experiencing disease reactivation following FTY withdrawal. Since the approval of FTY, other S1PRMs have been developed, including siponimod [38], ozanimod [15, 16], and ponesimod [39]. These newer agents offer improved selectivity for specific S1PR subtypes, potentially improving efficacy and safety profiles compared to FTY. While FTY is approved exclusively for RRMS, some newer S1PRMs have broader indications; siponimod, for instance, is also approved for active secondary progressive MS [38].

Despite its efficacy, some patients may need to discontinue FTY due to side effects, suboptimal response, or other reasons such as pregnancy planning [41]. A significant proportion of these individuals experience disease reactivation, sometimes referred to as “rebound” or “flare-up” [24, 25]. This phenomenon has gained increasing attention due to its critical clinical implications, highlighting the need for improved risk stratification and personalized management strategies in patients discontinuing FTY. Two potential mechanisms have been proposed in the literature: a massive egress of lymphocytes from lymphatic tissues as observed in a PLP139-151-immunized SJL mouse model [11], and astrocyte activation as revealed in an MS patient autopsy case [31]. However, the precise cellular and molecular drivers of such disease exacerbation remain incompletely understood.

Sex differences in MS extend beyond disease prevalence and also influence treatment response [43]. For instance, FTY has been shown to affect white blood cell counts more strongly in female patients, with grade 4 lymphopenia (according to Common Terminology Criteria of Adverse Events guidelines [52]) occurring more frequently in women than in men [2]. Although this sex-specific difference was not observed for grade 2 or 3 lymphopenia, the higher rate of severe lymphopenia in female patients has been associated with an increased risk of infections during the first year of FTY treatment [44, 64]. These findings underscore the existence of sex-dependent variations in immune response to S1PR modulation. However, only a limited number of studies have systematically addressed sex-specific differences in disease reactivation following S1PRM discontinuation.

We investigated sex-specific aspects of disease reactivation following FTY cessation in pwMS. Our analysis included patient cohorts from published literature as well as data from two open adverse event registries: the Adverse Event Reporting System (FAERS) operated by the US Food and Drug Agency (FDA) and EudraVigilance operated by the European Medicines Agency (EMA). To explore potential correlates of this phenomenon, we employed the experimental autoimmune encephalomyelitis (EAE) mouse model and assessed disease activity following treatment withdrawal. Tissue samples were processed histologically to assess S1PR1 and S1PR5 expression on infiltrating and perivascular T lymphocytes. CD3^+^T cells were selected as the primary cell population of interest given their predominance in MS lesion infiltrates [26] and their established role as the main target of FTY-mediated immune sequestration [48]. Our analyses consistently demonstrated that female sex is associated with higher disease reactivation risk following FTY withdrawal, with increased S1PR1 expression on CD3^+^T cells representing a potential cellular correlate of this sex-specific vulnerability.

## Materials and methods

### Literature research

To identify reports of disease reactivation following FTY withdrawal in pwMS, a systematic literature research was conducted on PubMed in accordance with PRISMA guidelines [55] (Supplementary Methods for more details). The following search terms were used “Rebound disease activation (or reactivation) Fingolimod”. Last update of the literature search was on January 2024. The following criteria had to be fulfilled for inclusion into the analysis: (i) published cohort study from 2010 (FTY approval) to 2024; (ii) inclusion of RRMS patients stopping FTY; (iii) inclusion of individuals who experienced disease reactivation (also referred to as ‘rebound’ according to each study’s definition) as well as individuals who did not exhibit any disease activity following FTY cessation, (iv) patients being older than 18 years of age. The reporting odds ratio (rOR) of disease reactivation after treatment cessation in female versus male patients was computed separately for each cohort, as well as for all cohorts together.

### Open registries analysis

Openly available spontaneously reported adverse event reports from the FDA FAERS [60] and EMA EudraVigilance [22] were analyzed. We downloaded all reports from FAERS (accessed March 5, 2024 via the FAERS public dashboard) and EudraVigilance (accessed March 5, 2024 via ADR reports [23]) for *Fingolimod*, *Fingolimod Hydrochloride*, and *Gilenya*. We only included monotherapy cases, i.e. only reports of cases where FTY is the only suspect drug. For FAERS data, we only included reports where no other substance was mentioned under *Suspect Product Active Ingredients.* For EudraVigilance data, we only included reports where no other drug classified as *suspect* was mentioned in *Suspect/interacting Drug List.* We additionally filtered reports by indication, retaining only those related to MS, using predefined MedDRA terminology search terms (see Supplementary Table S1). Although FTY is only approved in RRMS, this list contains more general terms to capture reports where the course is not nor not correctly reported. We then classified cases where *Rebound Effect* is mentioned under *Reactions* as rebound cases, and all other cases as no rebound and calculated rORs and their 95% confidence intervals (95% CI).

### Experimental autoimmune encephalomyelitis (EAE)

Animal studies were approved by the local authorities (Office of Agriculture and Nature, Bern, Switzerland: BE 142/20; external validation cohort: Niedersächsisches Landesamt für Verbraucherschutz und Lebensmittelsicherheit (Laves), Az 33.11.42502-04-19/3179).

C57BL/6JRj wild type (WT) mice were ordered from Janvier Labs (Le-Genest-Saint-Isle, France) and housed under conventional housing conditions at the in-house animal facility at Bern University.

Experimental autoimmune encephalomyelitis (EAE) was induced with myelin oligodendrocyte glycoprotein 35–55 (MOG_35-55_) peptide in 8 weeks old C57BL/6 mice of both sexes following our established protocol [1]; full procedural details are provided in the Supplementary Methods. Additionally, mice were treated from d0 to d19 post MOG_35–55_ EAE induction with 0.1 mg/kg FTY (Fingolimod, MedChem Express, South Brunswick, New Jersey, USA), diluted in condensed milk (MIGROS Kondensmilch, Migros, Bern, Switzerland). Condensed milk alone (no FTY) was given to control animals. This treatment was given orally by micropipette-guided drug administration [59]. Inclusion criteria are detailed in the Supplementary Methods.

Mice were weighed and scored for disease progression/severity or mobility impairment daily using a 10-point EAE scale by a blinded observer. The score was determined as follows: 0 = normal; 1 = reduced tail tone; 2 = flaccid tail paralysis; 3 = absence of reflex compensatory movements during walking; 4 = gait ataxia; 5 = slight paraparesis; 6 = moderate paraparesis or plegia of one leg; 7 = paraplegia with complete paralysis of both hindlimbs; 8 = tetraparesis; 9 = moribund; 10 = death. A mouse was considered experiencing disease reactivation after FTY cessation when its severity score was observed to be ≥ 2 points higher than on the last day of treatment (d19).

### Immunofluorescence of mice tissue

Paraffin-embedded spinal cord sections were heated at 60°C during 30 min. After rehydration, slides were placed in Tris–EDTA-Buffer (Trizma-Base (Sigma-Aldrich, 99.9%), 0.5 M EDTA pH 8 (Invitrogen), Tween20 (AppliChem), ddH2O) and heated 20 min in microwave (700W) to retrieve antigen sites. Tissues were blocked 1 h at RT with 10% NGS/PBS after application of Image-iT™ FX Signal Enhancer (Thermo, I36933). S1PR (rabbit-α-S1PR1 (Thermo, PA1-1040) or rabbit-α-S1PR5 (Thermo, PA5-98877) were co-stained with T cells (rat α-human CD3 (Bio-Rad, MCA1477). Goat-α-rabbit A647 (Thermo, A21245) and goat-α-rat A488 (Thermo, A11006) were used as secondary antibodies. Slides were mounted with DAPI Fluoromount-G (BioConcept, 0100-20). The specificity of the α-S1PR1 antibody was tested with corresponding blocking peptide (Thermo, PEP-220).

### Study cohort and neuropathological analysis of biopsied pwMS

Human brain tissue was obtained from pwMS experiencing rebound following FTY withdrawal, based on defined inclusion criteria (Supplementary Methods). Ethics approval was granted by the University Medical Center Göttingen (#19/09/10). Three female patients met inclusion criteria; five age- and sex-matched controls without prior S1PRM therapy were included. All biopsies were taken for differential diagnostics and not for study purposes.

Lesions were classified in two groups: active demyelinating (including early and late active lesions) and inactive demyelinated (detailed procedure in Supplementary Methods) according to established criteria [9] (Supplementary Table S2).

Immunofluorescent staining was used to assess inflammatory and receptor expression profiles (S1PR1, S1PR5 on CD3^+^T cells). S1PR1 was selected as the primary receptor of interest given its central role in lymphocyte egress and its well-established downregulation under FTY treatment [8]. S1PR5 was additionally assessed as it is also targeted by FTY and is expressed on specific lymphocyte subsets and oligodendrocytes relevant to MS pathophysiology [47]. Detailed staining protocol is provided in Supplementary Methods.

### Analysis of histological sections

Images were acquired using a Pannoramic 250 Flash III slide scanner (3DHISTECH) or Olympus VS200 slide scanner (human tissue). CD3^+^T cells were categorized as infiltrating or perivascular (examples in Supplementary Fig. S1). S1PRs staining intensity was determined using ImageJ (National Institute of Mental Health, NIH, Bethesda, USA). We quantified and reported the relative fluorescence intensity per cell based on CD3 surface staining (detailed description in Supplementary Methods).

### Statistical analysis

Statistical analyses were performed using GraphPad Prism (8.0.1), SPSS (IBM 25), and Python 3.9.7. Tests were selected based on cohort size (for larger cohorts [12, 28, 29, 46], we used Chi-square with Yates’s correction for continuity; or smaller cohorts [3, 24, 53, 58], we used Fisher’s Exact Probability test) and data structure, as specified in figure captions. Power analyses and bootstrapping were conducted using Python and G*Power 3.1.9.7. The reporting odds ratio (rOR) is the ratio of the reporting odds for rebound activity (the number of reports that mention rebound activity divided by the number of reports that do not) in female patients and the reporting odds for rebound activity in male patients; values above 1 indicate higher reporting odds in female patients, with statistical significance set at *p* < 0.05. Data are presented as mean ± standard error of the mean (SEM) or 95% CI; significance thresholds are indicated in figure and table legends. To account for multiple testing, we applied the Bonferroni correction. The adjusted significance levels are indicated in the corresponding figure and table legends. A full description of the statistical approach is available in the Supplementary Methods.

## Results

### Disease reactivation is associated with female sex in MS patients

**Fig. 1:**
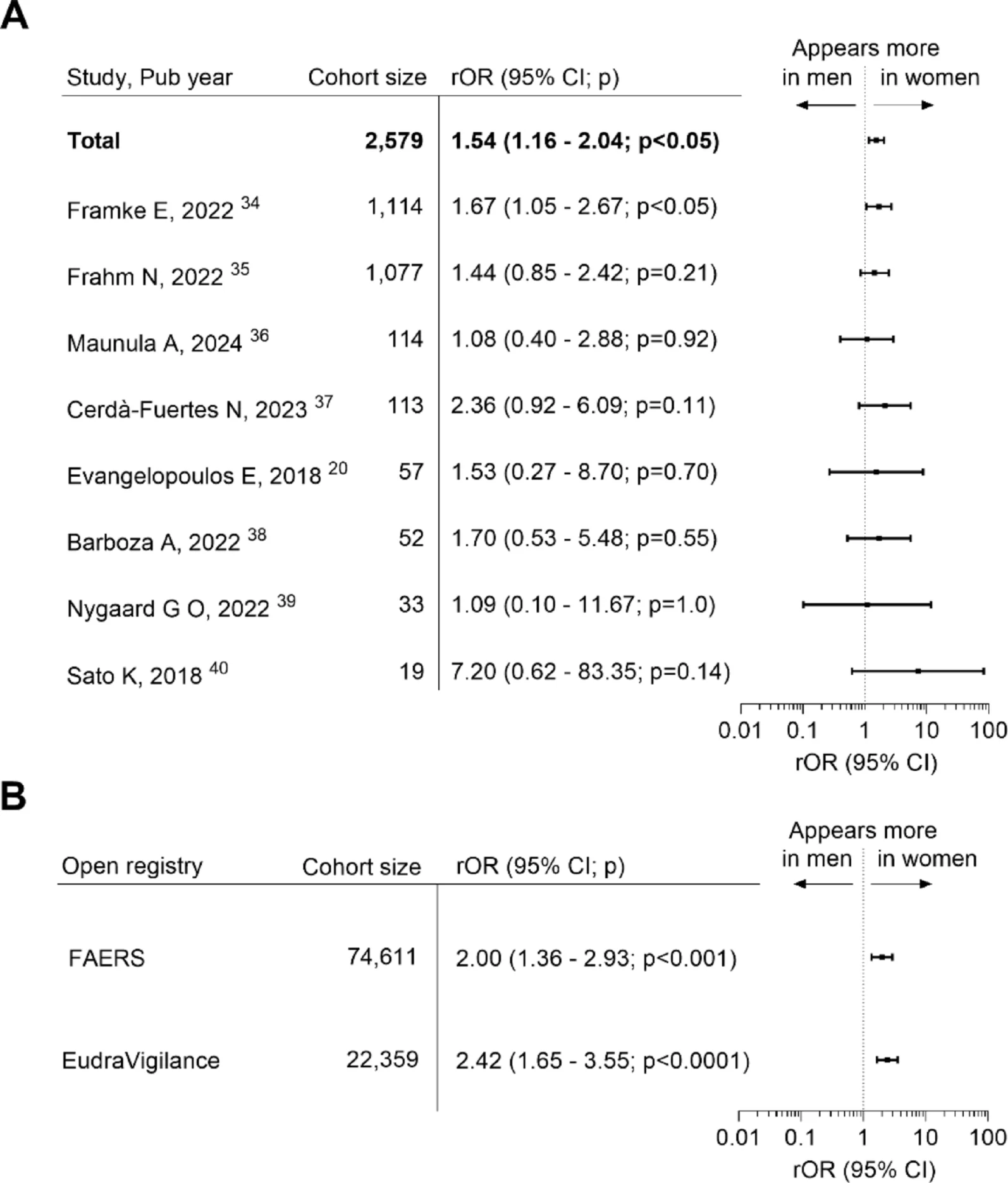
Disease reactivation (rebound) after FTY cessation is associated with female sex in MS patients. **A** Literature research performed on PubMed (as of January 25, 2024). Eight papers about disease reactivation after FTY cessation in pwMS were selected. rOR were calculated with 95% CI and corresponding *p*-value, based on sample size. **B** Analysis of FAERS and EudraVigilance for terms “rebound effect” in filtered reports with MS as main indication for FTY (monotherapy), as of March 5, 2024. These findings are statistically significant when accounting for multiple testing (Bonferroni). Abbreviations: CI: confidence interval, FAERS: FDA adverse event reporting system, FTY: fingolimod, rOR: reporting odds ratio, pwMS: people with multiple sclerosis

To address the question of sex-specific disease reactivation occurrence after drug cessation, a literature search was conducted on PubMed (Fig. 1A). Eight articles were selected, with a total of n = 2579 patients (Table 1). Altogether, we observed a pooled rOR of 1.54 (95% CI 1.16–2.04, *p* < 0.01), indicating higher MS reactivation risk in female compared to male pwMS.

**Table 1:** Number of reported cases of disease reactivation in literature

First author, year of publication	Disease reactivation		No disease reactivation	
	Female	Male	Female	Male
Framke E, 2022	100	24	706	284
Frahm N, 2022	77	19	724	257
Maunula A, 2024	25	7	63	19
Cerdà-Fuertes N, 2023	30	7	49	27
Evangelopoulos E, 2018	5	2	31	19
Barboza A, 2022	19	7	16	10
Nygaard G O, 2022	6	1	22	4
Sato K, 2018	9	1	5	4
TOTAL	271	68	1616	624

Studies not including cases without disease reactivation were excluded from the analysis and therefore not shown here. Additional demographic data such as age and geographical distribution were not consistently reported across the included studies and could therefore not be extracted

Furthermore, we analyzed open registries for adverse events concerning disease reactivation after FTY cessation (Fig. 1B). FAERS and EudraVigilance revealed n = 78,643 patient reports and n = 24,103 patient reports, respectively, after filtering for MS being the indication of medication, and FTY as monotherapy only (Table 2). This includes reports with unspecified sex. Among reports with specified sex, the number of rebound cases was 213 out of 58,053 female reports and 30 out of 16,315 male reports in FAERS; and 217 out of 16,571 female reports and 30 out of 5541 male reports in EudraVigilance. For the analysis shown in Fig. 1B, we used only the subset of reports in which patient sex was specified. Among sex-specified reports, females represented 78% of cases in FAERS and 75% in EudraVigilance, consistent with the known female predominance in MS [63]. Both registries displayed a significant association between female sex and disease reactivation (FAERS: rOR = 2.00, 95% CI 1.36–2.93, *p* < 0.0001; EV: rOR = 2.42, 95% CI 1.65–3.55, *p* < 0.0001).

**Table 2:** Number of reports with and without rebound effect for fingolimod as monotherapy. Only reports with known sex were considered in the analysis

Registry, sex	Report with Rebound	Reports without Rebound
EudraVigilance, female	217	16571
EudraVigilance, male	30	5541
EudraVigilance, sex unknown	18	1726
FAERS, female	213	58053
FAERS, male	30	16315
FAERS, sex unknown	39	3993

These convergent findings from independent clinical datasets consistently support a sex-specific vulnerability to disease reactivation following FTY withdrawal.

### Disease reactivation after FTY cessation in an MOG_35–55_ EAE mouse model

We investigated disease reactivation after FTY withdrawal in an MOG_35–55_ EAE mouse model, where disease reactivation was defined as an increase of 2 or more points in severity after treatment cessation (d20-d28).

We observed that 13 out of 38 female mice and 10 out of 50 male mice experienced a disease reactivation after treatment withdrawal (effect size (Cohen’s h, a measure of effect size for comparing two proportions) = 0.322, *p* = 0.15).

**Fig. 2:**
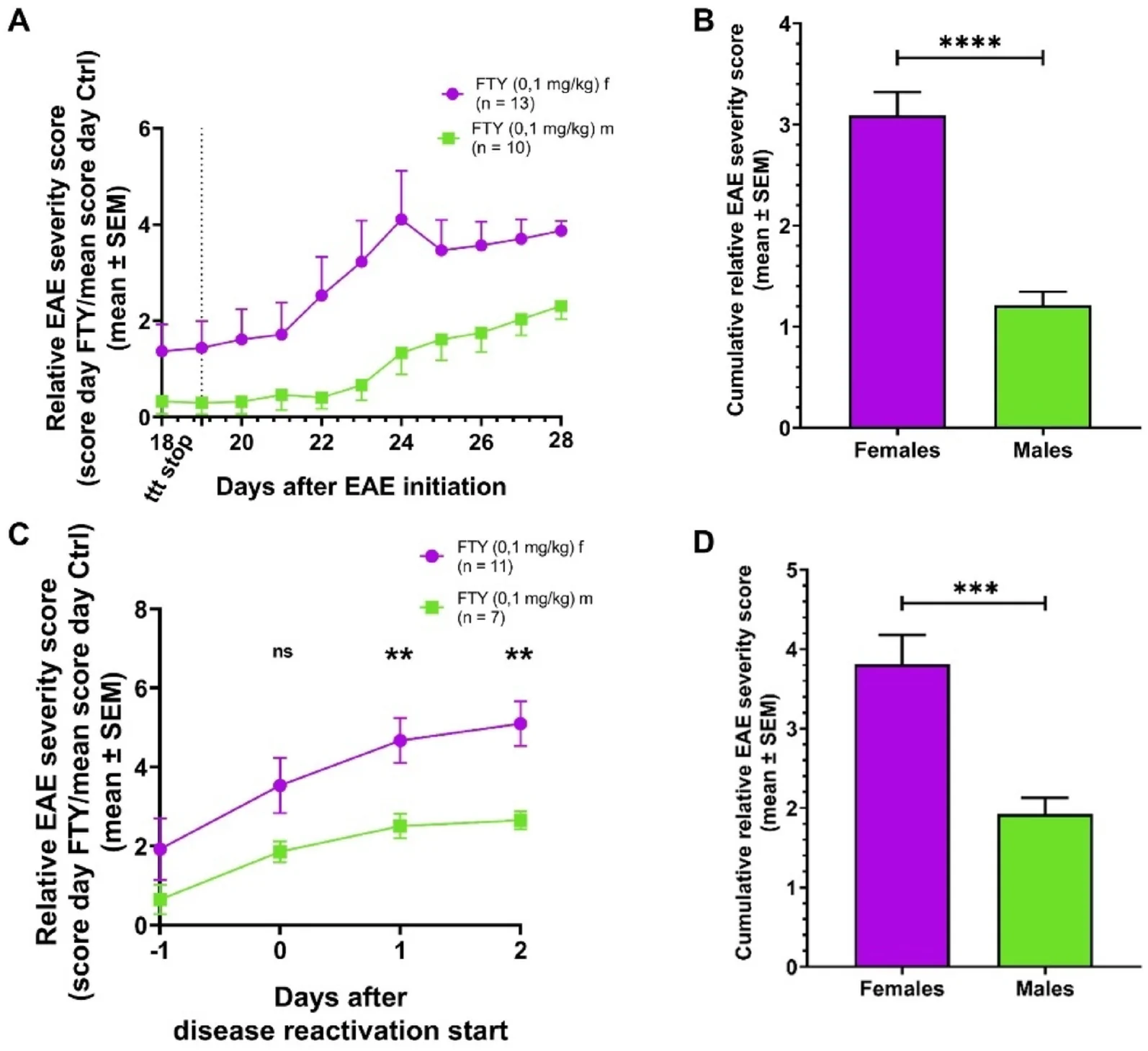
Female mice showed greater disease reactivation signs than male mice. **A** Clinical disease course of MOG_35-55_ EAE in WT mice relative to control mice (raw scores in Supplementary Fig. S2). Sexes are indicated in the graphs (f = female, m = male). Treatment (oral administration of FTY diluted in condensed milk, 0.1 mg/kg) was performed from d0-19. The scoring system is a 10-point scale. Numbers of animals are depicted in the graphs (only sick animals are shown). **B** Cumulative score (d20-28) quantification. **C** Disease reactivation onset was synchronized (corresponding to day 0 in the graph). Number of animals are depicted in the graph. **D** Cumulative score (d20-28) quantification after disease reactivation beginning synchronization. Abbreviations: d: day, EAE: experimental autoimmune encephalomyelitis, FTY: Fingolimod, MOG_35-55_: myelin oligodendrocyte glycoprotein peptide 35–55, SEM: standard error of the mean, WT: wild type. Statistics for panel B and D (d20-28): Mann–Whitney U test, ** < 0.01, *** < 0.001, **** < 0.0001

Regarding disease severity increase after treatment cessation at d20, we observed in control mice that females showed a significantly lower disease severity compared to males (Supplementary Fig. S2, *p* < 0.001). Therefore, scores of FTY-treated mice were normalized to the scores of control-treated mice of the respective sex. After normalization, we observed a greater disease severity in females compared to males during the post-treatment period (d20-28, 2.5-fold increase, *p* < 0.0001, Fig. 2A and B).

As mentioned above, disease reactivation onset was defined as an increase of ≥ 2 points in the clinical severity score relative to the last score recorded in the treatment period (d19). Based on this criterion, the reactivation onset was aligned across all animals to allow for comparative analysis. Following synchronization, we observed that, prior to and including reactivation onset, no statistically significant difference was observed regarding the severity of disease in female and male mice (Fig. 2C, days after disease reactivation start ≤ 0, *p* > 0.05). However, one and two days after onset, we observed that female mice experienced a more severe reactivation compared to male mice (both d1 and d2 *p* < 0.01). Additionally, the cumulative disease severity (d0 to d2 after onset) depicted a significant difference, with male mice being less affected in general compared to female mice (Fig. 2D, *p* < 0.0001).

These experimental findings corroborate the clinical observations, demonstrating that female sex is associated with more severe disease reactivation following FTY withdrawal in a controlled preclinical setting.

### Sex-specific regulation of S1PR1 but not S1PR5 following FTY treatment in MOG_35–55_ EAE Mice

**Fig. 3:**
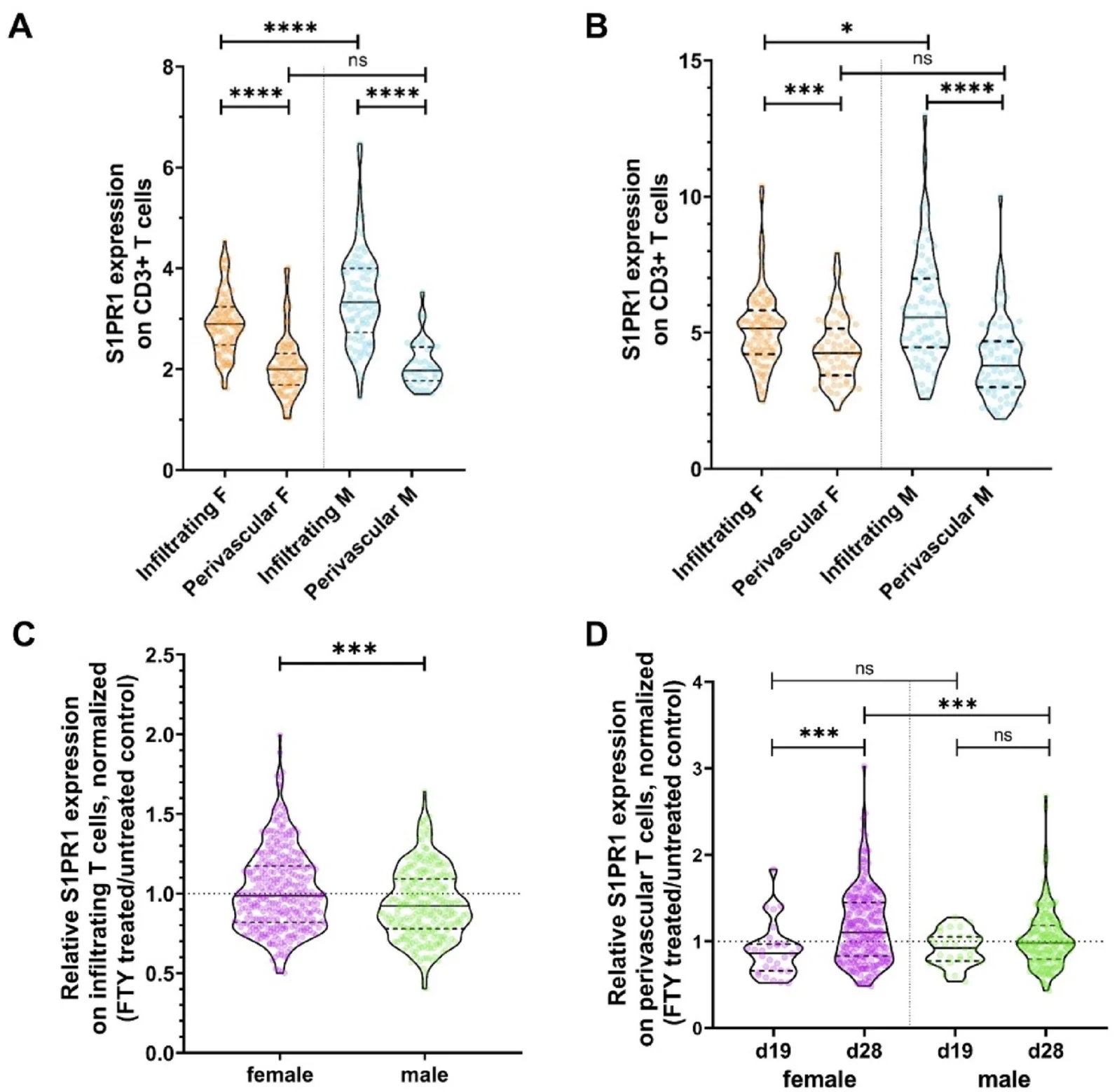
S1PR1 expression on spinal cord CD3^+^T cells across cohorts and treatment conditions. Immunofluorescence (IF) co-staining of S1PR1 and CD3 on paraffin sections of spinal cord lesions in MOG_35–55_ EAE mice. **A** Internal cohort without FTY treatment (d28). **B** External cohort (University Medical Center Göttingen) without FTY treatment. **C** Infiltrating CD3^+^T cells during disease reactivation after FTY withdrawal (d28). **D** Perivascular CD3^+^T cells in FTY-treated animals at various timepoints (d19 and d28). Dots represent individual cells. S1PR1 membrane intensity was normalized to local background, and in C–D further normalized to the mean of sex-matched control group (indicated by dotted line at y = 1). Medians are shown as solid black lines;first and third quartiles as dashed lines. Abbreviations: d: day, EAE: experimental autoimmune encephalomyelitis, FTY: fingolimod, IF: immunofluorescence, MOG₃₅–₅₅: myelin oligodendrocyte glycoprotein peptide 35–55, S1PR1: sphingosine 1-phosphate receptor 1. Statistics: Mann–Whitney U test; ns > 0.05, * < 0.05, *** < 0.001, **** < 0.0001. These findings are statistically significant when accounting for multiple testing (Bonferroni)

To investigate mechanisms triggering disease reactivation, immunohistochemistry was performed on MOG_35–55_ EAE spinal cord sections. In a first step, to ensure the reproducibility of our immunofluorescence findings, we analyzed an independent cohort of MOG_35–55_ EAE mice (untreated) provided through collaboration with the University of Göttingen. While FTY treatment could not be replicated, the staining patterns and sex-specific differences observed in this external validation cohort were consistent with our initial findings in control mice, independently validating our results and also accounting for 3Rs in animal welfare (Fig. 3A, B).

To assess the impact of FTY treatment on S1PR1 expression in CD3^+^T cells, we performed the staining in FTY treatment group and control (condensed milk alone) group (Supplementary Fig. S3–S5). After normalizing S1PR1 intensity on infiltrating CD3^+^T cells to the mean value of the control group of the respective sex, we found that S1PR1 expression in FTY-treated female mice was comparable to that of their respective controls, whereas FTY-treated male mice showed reduced expression compared to their respective controls (Fig. 3C). Furthermore, the changes from control in female and male mice differed significantly from each other (*p* < 0.001).

Similarly, relative S1PR5 expression on infiltrating CD3^+^T cells was assessed (Supplementary Fig. S6). Both male and female mice showed a significant decrease in relative receptor expression (f: 31.1%, *p* < 0.0001; m: 56.1%, *p* < 0.0001), with no statistically significant difference between the sexes (*p* > 0.05).

To better understand the impact of FTY on immune cell dynamics at the vascular interface, we specifically examined S1PR1 expression on perivascular CD3^+^T cells. FTY’s main mechanism of action is thought to be the trafficking modulation of immune cells, trapping cells into secondary lymphatic organs via decreased S1PR1 surface expression. Our histological analysis of perivascular CD3^+^T cells confirmed this in both female and male mice, showing a decreased S1PR1 expression under treatment (d19, no statistically significant sex differences (*p* > 0.05)) (Fig. 3D).

After treatment cessation, no statistically significant differences with control-treated mice were observed in male mice (d28, controls represented by y = 1 line, *p* > 0.05). However, FTY-treated female mice exhibited an increase of receptor expression on perivascular CD3^+^T cells, compared to control female (*p* < 0.001) and FTY-treated male mice (*p* < 0.001).

Together, these results identify sex-specific regulation of S1PR1 on CNS-infiltrating and perivascular CD3^+^T cells as a potential cellular correlate of the more severe disease reactivation observed in female mice.

### Increased S1PR1 expression in CD3^+^T cells in active demyelinating lesions of biopsies from pwMS

**Fig. 4:**
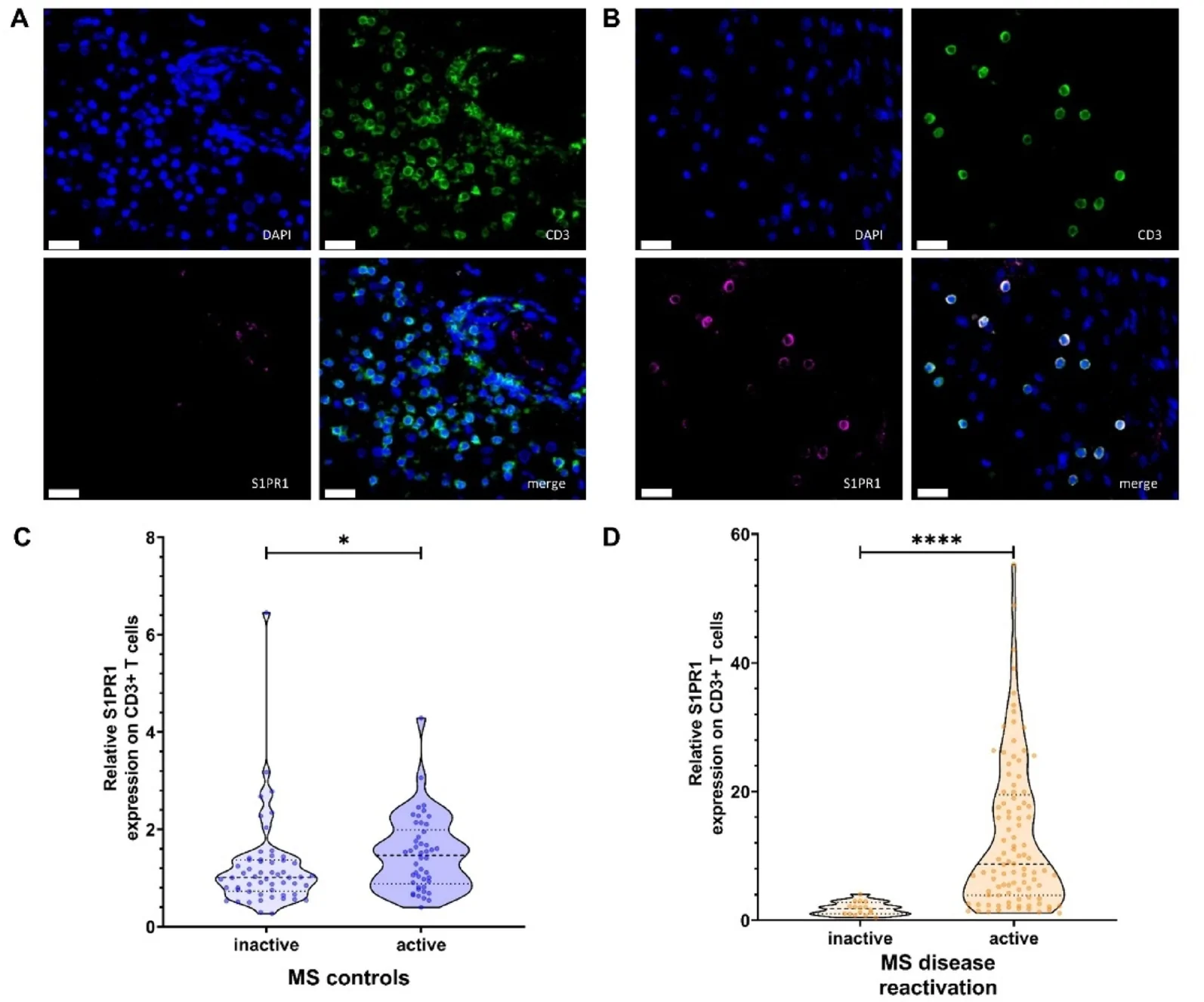
Increased S1PR1 expression on CD3^+^T cells in active demyelinating lesions in both MS controls and MS disease reactivation groups. **A** Representative image of immunofluorescent staining of a brain biopsy of an MS control patient (not treated with FTY) and **B** of a patient experiencing disease reactivation post-FTY cessation. Scale bars in A and B = 20 µm. **C** Quantification of S1PR1 expression levels on CD3^+^T cells in MS controls, demonstrating a significant increase in active demyelinating lesions (n = 3) compared to inactive demyelinated lesions (n = 2). **D** Quantification of S1PR1 expression levels on CD3^+^T cells in pwMS experiencing disease reactivation after FTY cessation. Increased S1PR1 expression in active demyelinating (n = 2) compared to inactive demyelinated (n = 1) lesions. One dot represents one cell. Medians are indicated by solid black horizontal lines; first and third quartiles are indicated by dashed black horizontal lines. Abbreviations: FTY: fingolimod, MS: multiple sclerosis, pwMS: people with MS, S1PR1: sphingosine 1-phosphate receptor 1. Statistics: Mann–Whitney U test, * < 0.05, **** < 0.0001

Extending beyond our findings in mice, we investigated whether similar mechanisms operate in human pathophysiology. A total of eight biopsies from female MS patients were analyzed (n = 5 pwMS controls (no S1PRM treatment); n = 3 MS disease reactivation cases after FTY cessation) (Supplementary Table S2 for characteristics and immunopatterns), comprising both active demyelinating and inactive demyelinated lesions. In both groups, S1PR1 expression on CD3^+^T cells was consistently higher in active demyelinating lesions compared to inactive demyelinated ones (MS controls: *p* < 0.05; disease reactivation: *p* < 0.0001) (Fig. 4). These results suggest that T cell-associated S1PR1 upregulation may be linked to the demyelinating activity, indicating lesion age, in MS lesions from female MS patients.

### Elevated S1PR1 expression in patients with disease reactivation compared to MS controls

**Fig. 5:**
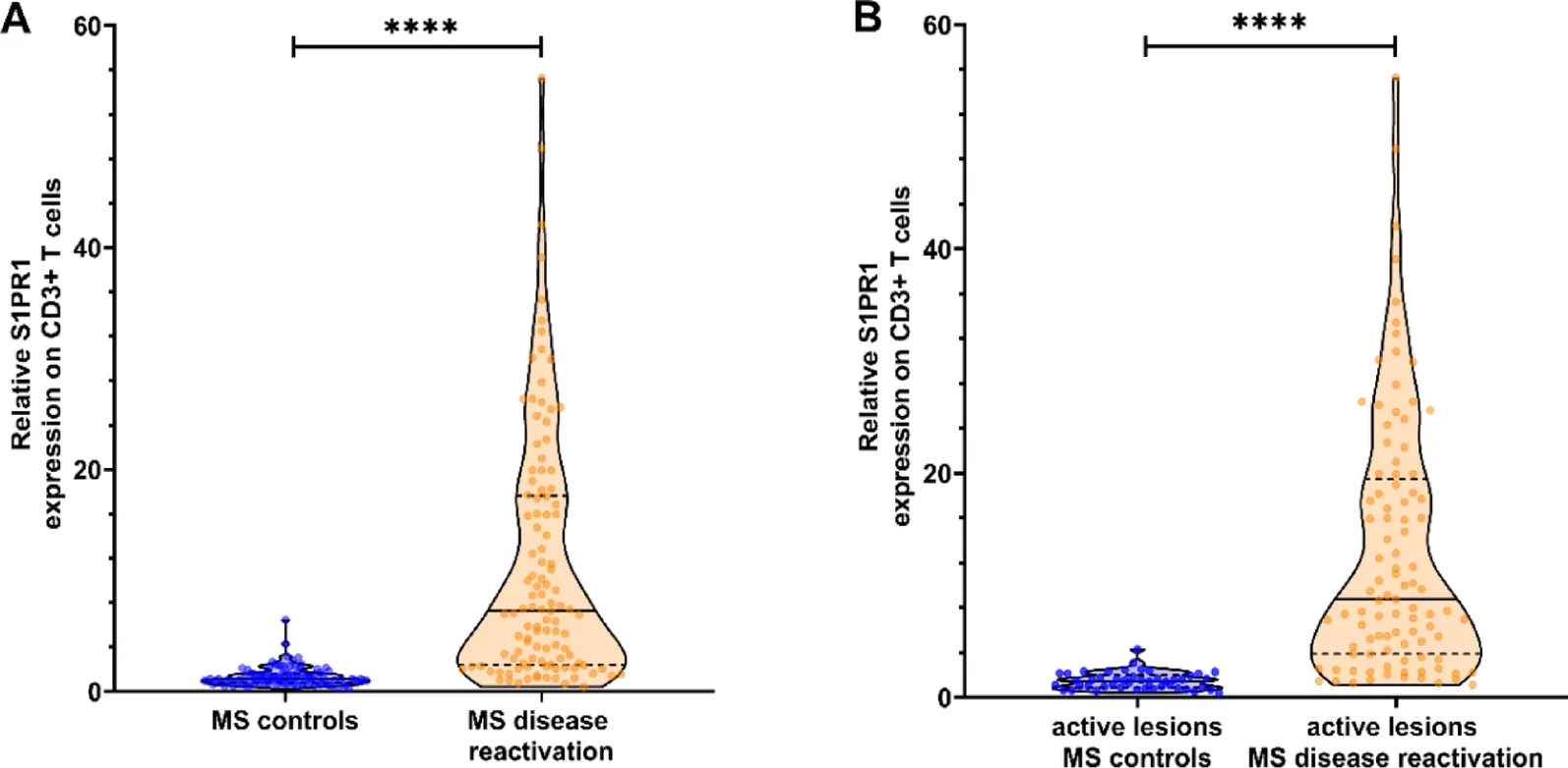
MS patients with disease reactivation showed higher S1PR1 expression on CD3^+^T cells compared to MS control patients. **A** Quantification of S1PR1 expression levels on CD3^+^T cells in MS controls (n = 5) compared to pwMS experiencing disease reactivation after FTY cessation (n = 3) (including both active demyelinating and inactive demyelinated lesions). **B** Quantification of S1PR1 expression levels on CD3+ T cells focusing on active demyelinating lesions (MS controls n = 3; MS disease reactivation n = 2). One dot represents one cell. Medians are indicated by solid black horizontal lines; first and third quartiles are indicated by dashed black horizontal lines**.** Abbreviations: FTY: fingolimod, MS: multiple sclerosis, pwMS: people with MS, S1PR1: sphingosine 1-phosphate receptor 1. Statistics: Mann–Whitney U test, **** < 0.0001

We next analyzed S1PR1 expression specifically in relation to clinical disease reactivation, comparing the disease reactivation group to MS controls (Fig. 5A). Interestingly, the S1PR1 expression was significantly higher in the disease reactivation group (*p* < 0.0001). These findings remained significant when only focusing on active lesions in cases and controls (Fig. 5B, *p* < 0.0001). Together, this reinforces a potential link between S1PR1-expressing CD3^+^T cells and the propensity for disease reactivation in female MS patients.

## Discussion

Across clinical, experimental, and neuropathological levels, our findings consistently associate female sex with more pronounced disease reactivation following FTY withdrawal. The convergent evidence from pharmacovigilance registries, EAE models, and human brain biopsies points toward sex-specific regulation of S1PR1 on CD3^+^T cells as a potential cellular correlate of this vulnerability, with a differential regulation of S1PR1 and S1PR5 suggesting receptor subtype-specific responses to treatment withdrawal.

The phenomenon of disease exacerbation following the discontinuation of immune cell trafficking inhibitors such as natalizumab and FTY is well recognized [4, 42, 49, 61], but the underlying mechanisms remain poorly characterized at the molecular level. Additionally, inconsistent terminology complicates comparisons across studies, with terms such as relapse, rebound, and disease reactivation often used interchangeably. In our investigations, the term “rebound” was used in open registries and literature analysis, ensuring that disease reactivation specifically following drug withdrawal—rather than relapses occurring during treatment—was analyzed. However, the need for standardized definitions remains.

In the present study, female sex was significantly associated with disease reactivation after FTY treatment cessation. This finding is consistent with the study by Framke et al. [29], which also reported a higher risk of disease reactivation in female patients after stopping FTY. However, sex differences are often not addressed in studies on disease reactivation. To minimize selection bias, we included a large cohort of patients from the German MS Register [28], which was not initially analyzed for sex-specific differences. Additionally, we examined open registries of adverse events to counteract selection effects. Although open adverse event registry analyses are inherently limited by incomplete metadata reporting, reporting bias, and limited medical expertise in consumer submitted reports, the large number of available reports enables a qualitative analysis of safety signals. Importantly, although pre- and post-treatment disease activity is not reported and a comparison of disease activity levels between sexes is therefore not possible, we specifically analyzed reports of rebound activity, i.e. reports that imply an improvement of symptoms under treatment and a worsening after cessation irrespective of baseline severity.

As previously reported, MS predominantly affects women, with a female-to-male ratio of 3:1 [34]. However, this disparity only becomes apparent after puberty [13], suggesting a role for sex hormones. Our results suggest that FTY may be more effective in male mice, as observed in our previous study [45]. Similarly, in the FREEDOMS trial, men treated with FTY were 86.7% disability-free (95% CI 80.7–92.8), whereas women were only 80.6% (95% CI 76.0–85.1) [21]. Additionally, male patients showed a greater reduction in the annual relapse rate when treated with FTY compared to placebo than female patients. These parallels between animal and human data support the presence of sex-specific differences in drug efficacy.

Sex-based differences in the immune system of MS patients may as well help explain our findings. Català-Senent et al. [10] reported that MS patients exhibit an altered immune system, with female patients tending to have a more pro-inflammatory environment and innate immune responses linked to the myeloid lineage, while male patients typically have adaptive responses associated with the lymphocyte lineage. However, the immune system is not only affected by the disease itself but also by FTY treatment. Previous studies have shown that FTY induces transcriptomic changes in lymphocytes [66], but it remains unclear whether these effects differ between sexes. FTY has also been shown to influence oxidative stress and directly modulate immune responses [51], both of which could contribute to disease reactivation upon withdrawal.

The sex-specific upregulation of S1PR1 observed in female mice after FTY withdrawal is particularly relevant given S1PR1’s critical role in lymphocyte egress from secondary lymphatic organs to inflammatory sites [8]. The increased S1PR1 expression on perivascular CD3^+^T cells in female mice may reflect enhanced lymphocyte trafficking to the CNS, contributing to more severe disease reactivation. This sex-specific upregulation of S1PR1 in female mice suggests an enhanced migratory potential of CD3^+^T cells compared to males following FTY withdrawal, potentially facilitating CNS infiltration. Sex hormones, particularly estrogen, have been shown to modulate S1PR1 expression and lymphocyte trafficking [5], which may partly underlie this differential response.

While our analysis focused on CD3^+^T cells (given their predominance in MS lesion infiltrates and their central role in S1PRM-mediated immune sequestration [26, 48]), we acknowledge that S1PR1 is also expressed on other immune cell populations, including B cells and monocytes [6]. Whether sex-specific differences in S1PR1 regulation extend to these compartments, particularly given the distinct reconstitution kinetics of CD19^+^B cells after FTY discontinuation [30], remains an open question for future studies.

Conversely, S1PR5 expression decreased in both sexes after FTY withdrawal. S1PR5 is primarily expressed on oligodendrocytes, natural killer cells, and certain T cell subsets, regulating cell survival, migration, and myelin homeostasis [47]. FTY acts as a functional antagonist on this receptor [18]. The relative balance between S1PR1 and S1PR5 expression appears to shift after treatment withdrawal, with a more pronounced increase toward pro-inflammatory S1PR1 predominance after FTY withdrawal. This imbalance may contribute to enhanced lymphocyte infiltration and neuroinflammation in females.

Despite similar overall susceptibility to EAE, male and female mice exhibit distinct immune responses that may influence disease progression and treatment outcomes. In the C57BL/6 strain, both sexes are equally susceptible to EAE induced by MOG injection, and no significant sex differences have been reported in the clinical course of the disease model [54, 56]. However, a recent study revealed differences at the molecular and cellular levels, despite similar overall EAE severity [65]. Specifically, female mice exhibited more pronounced spinal cord injury and demyelination, while male mice showed a stronger pro-inflammatory immune profile that was effectively counterbalanced by higher levels of anti-inflammatory and regulatory cell populations. This immunoregulatory balance in males may reduce inflammation and tissue damage, resulting in clinical scores comparable to those of females [65]. These observations align with our findings, suggesting that the sex-specific differences we observed in treatment response are rooted in intrinsic immune regulatory mechanisms. Whether similar findings would be observed in other models, such as the relapsing–remitting PLP139-151/SJL model [11], remains to be explored, as different antigens and genetic backgrounds may yield distinct immune dynamics.

Sex-based differences in immune function have been well established and likely contribute to the sex-specific treatment responses observed in our study. Females generally display heightened immune reactivity compared to males, due in part to the influence of sex hormones and the presence of two X chromosomes, which encode numerous immune-related genes [5]. This increased immune activation, while protective against infections, also predisposes females to a higher incidence of autoimmune diseases such as MS. Studies have demonstrated that X chromosome inactivation (XCI) is dynamically regulated in female T cells during immune activation and requires NF-κB signaling [27]. Importantly, several immune-relevant genes on the X chromosome, including Il2rg and Cxcr3, have been shown to escape XCI in T cells, contributing to increased gene dosage and enhanced immune signaling in females. Supporting this concept, the X-linked histone demethylase Kdm6a, which escapes X-inactivation, has been shown to promote neuroinflammation in EAE. Conditional deletion of Kdm6a in CD4^+^T cells or microglia reduces CNS inflammation in female mice, highlighting the contribution of X-linked immune regulators to sex differences in neuroinflammatory disease [35, 36, 62]. Although S1PR1 itself is not encoded on the X chromosome, these X-linked regulatory mechanisms may amplify immune signaling pathways that influence lymphocyte activation and migratory behavior. Thus, the increased S1PR1 expression observed in female mice after FTY withdrawal may reflect a broader sex-dependent amplification of immune responsiveness rather than a receptor-specific effect.

The mechanisms underlying post-FTY disease reactivation remain incompletely understood. In murine models, increased S1PR1 expression has been observed in lymphocytes sequestered within lymph nodes after drug withdrawal, facilitating their rapid release into circulation and potentially contributing to inflammation [11]. Consistent with these observations, our results revealed increased S1PR1 expression in CNS-infiltrating CD3^+^T cells of female EAE mice following FTY withdrawal, suggesting an enhanced migratory or activation phenotype in this setting.

The neuropathological findings extend these experimental observations to human pathology, demonstrating that S1PR1 upregulation on CD3^+^T cells is not only linked to lesion activity but is specifically elevated in women experiencing disease reactivation after FTY cessation. This provides rare direct translational evidence for receptor-level changes as a potential mechanism underlying sex-specific vulnerability. In our study, CD3 was used as a pan-T cell marker, and further subtyping into CD4^+^ and CD8^+^ populations was not performed. Based on published reconstitution kinetics, the CD3^+^T cells infiltrating the CNS during disease reactivation likely include a transient predominance of cytotoxic CD8^+^ cells, given that CD4^+^T cells and B cells require longer periods to recirculate [30, 33]. Whether these CD8^+^ cells display a heightened pathogenic or cytotoxic profile in females compared to males potentially amplified by sex-specific immune activation mechanisms [5] remains an important open question that future studies using T cell subset-specific markers should address.

Several hypotheses have been proposed to explain disease reactivation in humans, including the rapid resurgence of previously sequestered autoreactive lymphocytes following FTY withdrawal [4], while another posits that only specific lymphocyte populations contribute to this effect [19]. CD4^+^T cells are particularly susceptible to FTY treatment [48], followed by CD8^+^T cells [57]. Because CD4^+^T and CD19^+^B cells require longer periods to recirculate after FTY discontinuation [30, 33], a transient predominance of cytotoxic CD8^+^cells may occur, potentially contributing to acute CNS tissue damage. Within this framework, the increased S1PR1 expression observed on CD3^+^T cells in active lesions may reflect the dynamic recruitment of pathogenic lymphocyte populations during disease reactivation.

Our analysis was limited to female brain tissue, because biopsies from male pwMS with post-FTY disease reactivation were not available at the neuropathological laboratory. Consequently, the biopsy analysis was included to provide translational neuropathological evidence of S1PR1 upregulation during disease reactivation in human tissue, rather than to directly demonstrate sex differences in this component. While representing a bias, this also supports the overall sex distribution observed in our study. Accordingly, control cases were matched by sex and represent therefore female pwMS as well. Importantly, the availability of human CNS tissue from female patients experiencing post-FTY disease reactivation represents a unique strength of this study. Direct visualization of S1PR1 expression on infiltrating CD3^+^T cells within active demyelinating lesions provides rare translational evidence linking experimental observations to human pathology. When interpreted together with our murine and registry-based data, these findings strongly support the concept of a sex-associated vulnerability to disease reactivation following FTY withdrawal.

Previous studies have explored strategies to mitigate disease reactivation, including the use of dimethyl fumarate, which primarily reduces cytotoxic CD8^+^T cells. However, Delgado et al. [19] reported no significant effect in preventing post-FTY disease reactivation. Newer-generation S1P receptor modulators, with greater receptor specificity, may also help reduce the risk of disease reactivation. These findings highlight the need for further research to develop effective strategies for minimizing disease reactivation and identifying patients at greater risk for severe disease reactivation upon treatment cessation.

## Conclusion

Our study on clinical, experimental, and neuropathological data shows that female sex is consistently associated with a higher risk of disease reactivation following FTY cessation. In addition, across clinical data, experimental models, and human neuropathology, female sex was consistently associated with increased S1PR1 expression on CNS-infiltrating CD3^+^T cells. The concordant findings in female EAE mice and brain biopsies from female MS patients experiencing disease reactivation provide translational support for a sex-dependent role of S1PR1 in treatment withdrawal–associated disease activity. These findings highlight the need for further investigation into sex-specific differences in MS pathogenesis and treatment responses, as well as the development of personalized therapeutic strategies for MS patients.

## Supplementary Information

Below is the link to the electronic supplementary material.

Supplementary Material 1

## Data Availability

All data needed to evaluate the conclusions in the paper are present in the paper and/or the Additional file. Data from EudraVigilance are openly accessible online: [https://www.adrreports.eu/en/index.html].

## Abbreviations

ARR: Annualized relapse rate
CNS: Central nervous system
EAE: Experimental autoimmune encephalomyelitis
EMA: European medicines agency
FAERS: Food and drug administration adverse event reporting system
FDA: Food and drug administration
FTY: Fingolimod
MS: Multiple sclerosis
MOG: Myelin oligodendrocyte glycoprotein
PBMC: Peripheral blood mononuclear cells
pwMS: People with multiple sclerosis
RRMS: Relapsing–remitting multiple sclerosis
S1P: Sphingosine 1-phosphate
S1PR: Sphingosine 1-phosphate receptor
S1PRM: Sphingosine 1-phosphate receptor modulator
S1PR1: Sphingosine 1-phosphate receptor 1
S1PR5: Sphingosine 1-phosphate receptor 5
