# Supplementary material for: Higher disease reactivation risk in women after fingolimod withdrawal
Source: Acta Neuropathol Commun. 2026 Jun 17;14:179. doi: 10.1186/s40478-026-02343-6 (PMC13520175; doi:10.1186/s40478-026-02343-6)
Supplement: Supplementary file 1 [file 40478_2026_2343_MOESM1_ESM.docx]

Supplementary Material

**Sex Differences in Neurology: Higher Disease Reactivation Risk in Women after Fingolimod Withdrawal**

Massy M, Marti S, Kutllovci A, Frahm N, Fneish F, Ellenberger D, Hammer H, Chan A, Leichtle A, Lühder F, Metz I, Pistor M^*^, Hoepner R^*^

**Supplementary Methods**

**Supplementary Table S1. List of included MedDRA preferred terms for MS**

**Supplementary Table S2. Demographic, lesional, and treatment characteristics of MS patient biopsies**

**Supplementary Figure S1. Classification of CD3+ T cells based on tissue localization.**

**Supplementary Figure S2. MOG35-55 EAE mice are also affected by disease activation after FTY cessation**

**Supplementary Figure S3. Histological representation of CD3 and S1PR1 IF staining (DAPI counterstain) in lesion**

**Supplementary Figure S4. S1PR1 immunofluorescence blocking peptide**

**Supplementary Figure S5. Raw S1PR1 and S1PR5 expression data on infiltrating CD3+ T cells immunofluorescence**

**Supplementary Figure S6. Effect of FTY treatment on S1PR5 expression on infiltrating CD3+ T cells of MOG35-55 EAE mice**

**Supplementary Methods**

**Literature research**

Studies were excluded if the paper was only reporting disease reactivation. One reviewer screened the articles for selection, based on these above-mentioned criteria. From the selected articles, we extracted the following data: first author, year of publication, total number of patients, number of female/male patients, and number of disease reactivation cases after FTY cessation for each sex. If necessary, we reached out to the corresponding authors of the relevant papers to obtain clarification on missing data, instances where data were not reported in the paper, or cases where data were presented in an unconventional manner.

**Experimental autoimmune encephalomyelitis (EAE)**

Eight weeks old C57BL/6 mice of both sexes were immunized with an emulsion of myelin oligodendrocyte glycoprotein peptide 35-55 (MOG_35-55_, Institute of Medical Immunology, Charité, Berlin) in PBS (Dulbecco’s Phosphate Buffered Saline, Biowest, L0615-500) and resuspended in an equal volume of Complete Freund’s Adjuvant (CFA) (Incomplete Freund’s Adjuvants (Santa Cruz, sc24648) and 100 µg heat inactivated Mycobacterium tuberculosis (Difco 231141)). A subcutaneous injection of 100 µg MOG_35-55_/CFA was performed after 5% Isoflurane anesthesia (2ml/min). Two intraperitoneal injections of 200 ng Pertussis Toxin (List Biological Labs, 180) were given on d0 and d2. This part of the study investigates disease reactivation following treatment cessation in a murine model of EAE. Animals were included based on two predefined criteria: (i) the development of clinical EAE (score ≥2/10), and (ii) a reactivation event defined as an increase of ≥2 points in clinical score after the final day of treatment (day (d) 19). Non-sick animals as well as animals not showing disease reactivation signs (2 males and 1 female) were excluded from the analysis. Thirteen female and ten male animals met these criteria and were included in the analysis. For time-aligned disease reactivation analysis (e.g., Fig. 2C-D), two female and three male animals were excluded due to insufficient follow-up (<3 days post-reactivation).

In a previous study focused on treatment efficacy, a subset of the mice (n=8) was analyzed during the treatment phase (d0 to d19), including both symptomatic and asymptomatic mice31. In contrast, the present work focuses exclusively on the post-treatment phase (d20 to d28) in animals experiencing clinical reactivation. In additional experiments, a proportion of animals were sacrificed at d19 for endpoint tissue collection and immunostaining. These animals did not contribute to the EAE score reactivation dataset, as they were not monitored beyond the treatment phase. In line with the principles of the 3Rs (Replacement, Reduction, and Refinement), previously collected data from in total 8 mice were reused where applicable, minimizing animal use while maintaining scientific integrity.

**Study cohort of biopsied MS**

The study of human brain tissue from pwMS was approved by the Ethics Committee of the University Medical Center Göttingen (#19/09/10) and included formalin-fixed, paraffin-embedded archival tissue from pwMS with the following inclusion criteria: (i) biopsy showing an inflammatory demyelinating disease consistent with MS, (ii) pwMS experiencing a rebound after S1PRM withdrawal that led to biopsy, (iii) sufficient tissue available for further analyses. We identified three female patients meeting the inclusion criteria (of a database containing more than 1,000 patients); no male patient met the inclusion criteria. As a control group, we identified five sex-matched (female) patients with a similar age at biopsy and comparable lesional activity with no prior S1PRM therapy.

All biopsies were taken for differential diagnostics and not for study purposes.

**Neuropathological analysis of human brain biopsies**

Formalin-fixed, paraffin-embedded brain tissue sections were stained with hematoxylin and eosin, luxol fast blue/periodic acid-Schiff, and Bielschowsky’s silver impregnation. Immunohistochemistry was performed using antibodies against key myelin proteins, including myelin basic protein (MBP), proteolipid protein (PLP), myelin oligodendrocyte glycoprotein (MOG), 2′3′-cyclic nucleotide 3′-phosphodiesterase (CNP), and myelin-associated glycoprotein (MAG) (described in detail by Stork et al.^1^). Lesions were assessed by at least one board-certified neuropathologists (I.M.) and classified according to established criteria^2^ (Supplementary Table 2). Early active lesions were identified by macrophages containing both major (MBP, PLP) and minor (CNP, MOG, MAG) myelin proteins, while late active lesions showed immunoreactivity only for major myelin proteins. Inactive demyelinated lesions lacked detectable myelin proteins in macrophages. These categories reflect different stages in the demyelination process, with early active lesions representing the most recent activity. For analytical purposes, lesions were stratified into two groups: active demyelinating (including early and late active lesions) and inactive demyelinated, due to the limited number of cases within individual subtypes.

Following lesion stratification, additional immunofluorescent staining was performed to characterize inflammatory and receptor expression profiles within the same tissue sections. Tissue sections were first deparaffinized by heating at 60°C for 1 hour and rehydrated. Antigen retrieval with Tris–EDTA (pH 9.0) was employed to unmask epitopes. After incubation for 30–60 minutes at RT in blocking buffer (10% NGS/PBS), slides were incubated overnight at 4 °C with the primary antibody anti-CD3 (1:50, MCA1477, Bio-Rad). The next day, sections were washed three times with PBS and the fluorescent secondary antibody (Alexa Fluor 647 anti-rat, 1:100) was applied (100 µL per section) and incubated for 1 hour at RT, followed by PBS washes. After a second blocking step for 20-30 minutes, additional staining with anti-S1PR1 (1:400, ab233386, abcam) was performed. On the following day, the sections were rinsed with PBS before applying the fluorescent secondary antibody (Cy3 anti-rabbit, 1:100). Incubation was carried out at RT for 1 hour, after which the sections were washed with PBS. Nuclear counterstaining was carried out with DAPI (final concentration 1:10,000) for 15 minutes at RT. Sections were washed with PBS, transferred to distilled water, and mounted with Dako Fluorescence Mounting Medium (S3023, Agilent).

**Analysis of histological sections**

CD3+ T cells were classified into two categories (infiltrating or perivascular) based on their localization in the tissue (see Supplementary Fig. 1 for example). We quantified and reported the relative fluorescence intensity per cell based on CD3 surface staining.

S1PRs staining intensity was determined as followed: T cells marked by CD3 positive staining were selected by a macro based on the fluorescence intensity (intensity threshold). Each cell was trimmed, the background was removed and the fluorescence intensity for S1P receptor 1 or 5 was obtained for the whole cell, as well as the nucleus, the cytoplasm and the membrane (starting delineation with 3 pixels less). The fluorescence intensity of a surrounding region (3 pixels away from the membrane) to each cell was obtained to normalize to local background

**Statistical analysis**

Analyses were performed with GraphPad Prism version 8.0.1 and SPSS (IBM SPSS statistics 25).

For each patient cohort in the literature research, the rOR and its 95% CI was calculated. The method used for calculation of the corresponding p-value was determined based on sample size; for larger cohorts^3–6^, we used Chi-square with Yates’s correction for continuity. For smaller cohorts^7–10^, we used Fisher’s Exact Probability test.

To analyze open registry data, we used Python 3.9.7 with the packages numpy and pandas for data preprocessing, and scipy.stats for bootstrapping.

For the power calculations, we used G*Power 3.1.9.7^11^ and Python 3.9 with the packages numpy, pandas, matplotlib, seaborn, and scipy.stats.

Mann-Whitney U test was used when comparing 2 groups. The statistical tests used in each figure are indicated in figure captions. To analyze disease reactivation in the animal experiments after treatment stop, statistics were conducted from day 20 (d20) to day 28 (d28).

Data are represented as mean values ± standard error of the mean (SEM) or 95% CI. The level of significance is indicated as followed: ns (not significant): p-value > 0.05, *: p-value < 0.05, **: p-value < 0.01, ***: p-value < 0.001 and ****: p-value < 0.0001. To account for multiple testing, we applied the Bonferroni correction. The adjusted significance levels are indicated in the corresponding figure and table legends.

**References**

1.	Stork L, Ellenberger D, Beißbarth T, et al. Differences in the reponses to apheresis therapy of patients with 3 histopathologically classified immunopathological patterns of multiple sclerosis. JAMA Neurol. 2018;75:428–435.

2.	Brück W, Porada P, Poser S, et al. Monocyte/Macrophage Differentiation in Early Multiple Sclerosis Lesions Materials and Methods Biopsy Material and Clinical Data. Ann Neurol. 1996;38:788–796.

3.	Framke E, Pontieri L, Bramow S, Sellebjerg F, Magyari M. Rebound of clinical disease activity after fingolimod discontinuation? A nationwide cohort study of patients in Denmark. J Neurol Neurosurg Psychiatry. 2022;93:1317–1321.

4.	Frahm N, Fneish F, Ellenberger D, et al. Therapy Switches in Fingolimod-Treated Patients with Multiple Sclerosis: Long-Term Experience from the German MS Registry. Neurol Ther. Springer Healthcare; 2022;11:319–336.

5.	Maunula A, Atula S, Laakso SM, Tienari PJ. Frequency and risk factors of rebound after fingolimod discontinuation – A retrospective study. Mult Scler Relat Disord [online serial]. Elsevier B.V.; 2024;81:105134. Accessed at: https://doi.org/10.1016/j.msard.2023.105134.

6.	Cerdá-Fuertes N, Nagy S, Schaedelin S, et al. Evaluation of frequency, severity, and independent risk factors for recurrence of disease activity after fingolimod discontinuation in a large real-world cohort of patients with multiple sclerosis. Ther Adv Neurol Disord. 2023;16.

7.	Evangelopoulos ME, Miclea A, Schrewe L, et al. Frequency and clinical characteristics of Multiple Sclerosis rebounds after withdrawal of Fingolimod. CNS Neurosci Ther. 2018;24:984–986.

8.	Barboza A, Gaitán MI, Alonso R, et al. Rebound activity after fingolimod cessation: A case – control study. Mult Scler Relat Disord. 2022;57.

9.	Nygaard GO, Torgauten H, Skattebøl L, et al. Risk of fingolimod rebound after switching to cladribine or rituximab in multiple sclerosis. Mult Scler Relat Disord. 2022;62.

10.	Sato K, Niino M, Kawashima A, Yamada M, Miyazaki Y, Fukazawa T. Disease exacerbation after the cessation of fingolimod treatment in japanese patients with multiple sclerosis. Intern Med. 2018;57:2647–2655.

11.	Faul F, Erdfelder E, Lang AG, Buchner A. G*Power 3: A flexible statistical power analysis program for the social, behavioral, and biomedical sciences. Behav Res Methods. 2007;39:175–191.

**Supplementary Table S1. List of included MedDRA preferred terms for MS**

*Terms*

Multiple sclerosis

Clinically isolated syndrome

Marburg's variant multiple sclerosis

Multiple sclerosis pseudo relapse

Multiple sclerosis relapse

Primary progressive multiple sclerosis

Progressive multiple sclerosis

Progressive relapsing multiple sclerosis

Radiologically isolated syndrome

Relapsing multiple sclerosis

Relapsing-remitting multiple sclerosis

Secondary progressive multiple sclerosis

Tumefactive multiple sclerosis

**Supplementary Table S1**: List of included MedDRA preferred terms for multiple sclerosis (MS).

**Supplementary Table S2. Demographic, lesional, and treatment characteristics of MS patient biopsies**

| **MS Controls** | | | | |  |
| --- | --- | --- | --- | --- | --- |
| *ID* | *Age group* | | *Sex* | *Lesional activity* | *Treatment* |
| P1 | 40-49 | | female | early active demyelinating, IP II | relapse treatment (high dose steroids) |
| P2 | 40-49 | | female | early active demyelinating, IP II | no treatment before biopsy, although MS was known |
| P3 | 30-39 | | female | inactive demyelinated | relapse treatment (high dose steroids) before biopsy, as well as interferon-beta and mitoxantrone |
| P4 | 30-39 | | female | inactive demyelinated | none |
| P5 | 40-49 | | female | early active demyelinating, IP I | relapse treatment (high dose steroids) |
|  |  |  | |  |  |
| **MS Fingolimod Disease Reactivation Cases** | | | | |  |
| *ID* | *Age group* | | *Sex* | *Lesional activity* | *Treatment* |
| P6 | 20-29 | | female | early active demyelinating, NMO like features, IP II and III features | FTY stopped 11 weeks before biopsy, relapse treatment before biopsy (high dose steroids) |
| P7 | 30-39 | | female | inactive demyelinated | FTY stopped 13 weeks before biopsy, relapse treatment after biopsy (high dose steroids) |
| P8 | 20-29 | | female | early and late active demyelinating, IP I | FTY stopped 15 weeks before biopsy, relapse treatment before biopsy (high dose steroids) |

**Supplementary Table S2:** Demographic, lesional, and treatment characteristics of MS patient biopsies. Ages were grouped into 10-year intervals to protect participant confidentiality. Abbreviations: FTY: fingolimod, IP: immunopattern, MS: multiple sclerosis, NMO: neuromyelitis optica.

**Supplementary Figure S1.** **Classification of CD3+ T cells based on tissue localization.**

**Supplementary Figure S1: Classification of CD3+ T cells based on tissue localization.** Representative image of control MOG_35-55_ EAE mice spinal cord staining distinguishing perivascular and infiltrating CD3+ T cells. CD3 (AF488) and S1PR1 (A647) IF staining (DAPI counterstain) in lesion. Perivascular T cells are defined as those clustered immediately adjacent to blood vessels, without evidence of migration into the surrounding parenchyma. In contrast, infiltrating T cells are observed dispersed within the tissue parenchyma, distant from vascular structures. Scale bars = 0.5 mm. Abbreviations: EAE: experimental autoimmune encephalomyelitis, MOG_35-55_: myelin oligodendrocyte glycoprotein 35-55 peptide, S1PR1: sphingosine 1-phosphate receptor 1.

**Supplementary Figure S2. MOG_35-55_ EAE mice also experience disease activation after FTY cessation**

Supplementary Figure S2: MOG_35-55_ EAE mice also experience disease activation after FTY cessation. Clinical disease course of MOG_35-55_ EAE in WT mice. Sexes are indicated in the graphs (f=female, m=male). Left panel: Control group (no FTY, condensed milk alone). Right panel: Treatment group (d0-d19, oral administration of FTY diluted in condensed milk at dose of 0.1 mg/kg). The EAE score system is a 10-point scale. Numbers of animals are depicted in the graphs. Abbreviations: EAE: experimental autoimmune encephalomyelitis, FTY: fingolimod, MOG_35-55_: myelin oligodendrocyte glycoprotein peptide 35-55, SEM: standard error of the mean, WT: wild type. Statistics (d20-d28): Mann-Whitney U test, ** < 0.01, ***: p-value < 0.001.

**Supplementary Figure S3. Histological representation of CD3 and S1PR1 IF staining (DAPI counterstain) in lesion**

**Supplementary Figure S3:** Histological representation of CD3 and S1PR1 IF staining (DAPI counterstain) in lesion. Scale bars = 20µm. Abbreviations: IF: immunofluorescence, S1PR1: sphingosine 1-phosphate receptor 1.

**Supplementary Figure S4. S1PR1 Immunofluorescence blocking peptide**

**Supplementary Figure** **S4**: S1PR1immunofluorescence blocking peptide. Histological representation of CD3 and S1PR1 IF staining (DAPI counterstain) of control (no FTY, condensed milk only) mouse, compared to immunostaining with corresponding blocking peptide (PEP-220, Invitrogen, according to manufacturer’s instructions). Scale bars = 100µm. Abbreviations: FTY: fingolimod, IF: immunofluorescence, S1PR1: Sphingosine 1-phosphate receptor 1.

**Supplementary Figure S5. Raw S1PR1 and S1PR5 expression data on infiltrating CD3+ T cells immunofluorescence quantification**

**Supplementary Figure** S**5**: Raw data of immunofluorescent staining quantification of (A) S1PR1 and (B) S1PR5 on infiltrating CD3+ T cells. Female (F) and male (M) cells are depicted in orange/purple and blue/green, respectively. Control=no FTY, condensed milk only; Treated= FTY 0.1 mg/kg in condensed milk. Medians are indicated by solid black horizontal lines; first and third quartiles are indicated by dashed black horizontal lines**.** Abbreviations: FTY: fingolimod, S1PR1/5: sphingosine 1-phosphate receptor 1/5. Statistics: Mann-Whitney U test ns > 0.05, * < 0.05, *** < 0.001, **** < 0.0001.

**Supplementary Figure S6. Effect of FTY treatment on S1PR5 expression on infiltrating CD3+ T cells of MOG_35-55_ EAE mice**

**Supplementary Figure S6:** Effect of FTY treatment on S1PR5 expression on infiltrating CD3+ T cells of MOG_35-55_ EAE mice. A) Histological representation of CD3 and S1PR5 IF staining (DAPI counterstain) in lesion. Scale bars = 20µm. B) Immunofluorescence quantification. Dots represent cells found in spinal cord lesion of sick animals experiencing disease reactivation after FTY treatment cessation. S1PR5 intensity was normalized first to local surrounding background, second with the mean intensity of the control group (indicated by y=1 dotted line) of the respective sex (see Supplementary Fig. 5 for raw data). Medians are indicated by solid black horizontal lines; first and third quartiles are indicated by dashed black horizontal lines. Abbreviations: CI: confidence interval, EAE: experimental autoimmune encephalomyelitis, FTY: fingolimod, IF: immunofluorescence, MOG_35-55_: myelin oligodendrocyte glycoprotein 35-55 peptide, S1PR5: sphingosine 1-phosphate receptor 5. Statistics: Mann-Whitney U test, ns > 0.05.
